# From Quantum Probabilities to Quantum Amplitudes

**DOI:** 10.3390/e22121389

**Published:** 2020-12-08

**Authors:** Sofia Martínez-Garaot, Marisa Pons, Dmitri Sokolovski

**Affiliations:** 1 Departamento de Química-Física, Universidad del País Vasco, UPV/EHU, 48940 Leioa, Spain; sofia.martinez@ehu.eus; 2 Departamento de Física Aplicada I, Universidad del País Vasco, UPV-EHU, 48013 Bilbao, Spain; marisa.pons@ehu.eus; 3 IKERBASQUE, Basque Foundation for Science, 48011 Bilbao, Spain

**Keywords:** quantum measurements, Pauli problem, transition amplitudes, weak measurements, quantum particle’s past

## Abstract

The task of reconstructing the system’s state from the measurements results, known as the Pauli problem, usually requires repetition of two successive steps. Preparation in an initial state to be determined is followed by an accurate measurement of one of the several chosen operators in order to provide the necessary “Pauli data”. We consider a similar yet more general problem of recovering Feynman’s transition (path) amplitudes from the results of at least three consecutive measurements. The three-step histories of a pre- and post-selected quantum system are subjected to a type of interference not available to their two-step counterparts. We show that this interference can be exploited, and if the intermediate measurement is “fuzzy”, the path amplitudes can be successfully recovered. The simplest case of a two-level system is analysed in detail. The “weak measurement” limit and the usefulness of the path amplitudes are also discussed.

## 1. Introduction

The Pauli problem [1] questions the possibility of deducing the theoretical quantum state (wave function) from the observed statistics of quantum measurements. The measurements are assumed to be ideal (i.e., infinitely accurate), and a reconstruction of the state, if at all possible, requires measuring several non-commuting operators (see for example [2,3,4,5,6,7] and Refs. therein).

Below we will consider the problem in a somewhat broader context. Quantum mechanics predicts probabilities of the outcomes of series of consecutive measurements by defining probability amplitudes for virtual histories (Feynman paths), followed by the system. The path amplitudes are then added, as appropriate, and the absolute square of the sum gives the probability of a particular scenario to occur [8]. The measurements usually considered in connection with the Pauli problem are, in fact two-step sequences, consisting of preparing the system in an initial state and measuring the chosen variable later. The case of a preparation followed by two or more measurements made on the system is richer, since it allows for a different type of interference not available to the two-step histories.

In this paper we consider a different “Pauli problem”, namely the possibility of recovering the system’s path amplitudes from the results of intermediate fuzzy measurements, and discuss how it can be done in practice. The rest of the paper is organised as follows. In Section 2 we revisit the basic rules for constructing probabilities with the help of virtual (Feynman) paths. In Section 3 we apply the recipe of Section 2 to a composite *system* + *probe*, and explain why the two-step histories are insufficient for our purpose. In Section 4 we extend the approach to three-step histories where a system is “pre- and post-selected” in known initial and final states. In Section 5 and Section 6 we apply the method to the simplest case of a two-level system (a qubit), and in Section 7 provide a numerical simulation. Section 8 discusses the “strong” and “weak” limiting cases where the approach of Section 4, Section 5, Section 6 and Section 7 fails, and analyses the reason for the failure. In Section 9 we evaluate the “strong” and “weak” averages of the pointer’s readings, and briefly comment on the popular subject of the so-called “weak measurements” [9]. Usefulness of the paths amplitudes for predicting the outcomes of future measurements, and making statements about the system’s past is analysed in Section 10. Section 11 contains our conclusions.

## 2. From Amplitudes to Probabilities

We start by recalling the rules for evaluating the probabilities of three consecutive measurements, given in [8], and recently revisited in [10,11,12]. Consider such measurements performed on a quantum system (*s*), with which one associates a *N*-dimensional Hilbert space, $H^{s}$. Three quantities, represented by operators ${\hat{Q}}^{\ell }$, ℓ=0,1,2, are measured at t=0, $t=t^{\prime }>0$, and $t=t^{\prime \prime }>t^{\prime }$, respectively. Each operator has $M_{\ell }\leq N$ possibly degenerate eigenvalues, $Q_{m_{\ell }}^{\ell }$, $m_{\ell }=1,\cdots ,M_{\ell }$, and can be written as

(1) $${\hat{Q}}^{\ell }=\sum_{m_{\ell }=1}^{M_{\ell }}Q_{m_{\ell }}^{\ell }\sum_{n_{\ell }=1}^{N}\Delta \left(Q_{m_{\ell }}^{\ell }-\langle q_{n_{\ell }}^{\ell }\left|{\hat{Q}}^{\ell }\right|q_{n_{\ell }}^{\ell }\rangle \right)\left|q_{n_{\ell }}^{\ell }\rangle \langle q_{n_{\ell }}^{\ell }\right|,$$

where $|q_{n_{\ell }}^{\ell }\rangle$, $n_{\ell }=1,\cdots ,N$ form a suitable orthonormal basis, and Δ(x−y)≡1 for x=y, and 0 otherwise.

**Figure 1:**
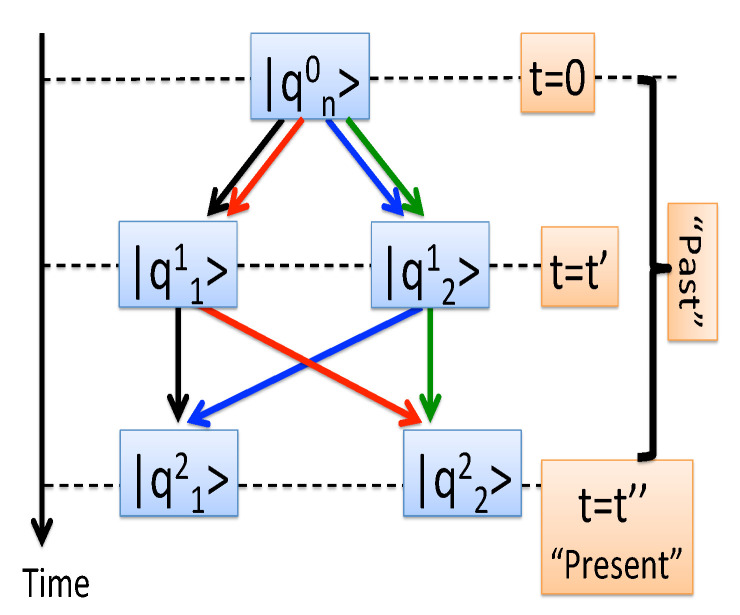
Four virtual paths for a two-level system. The times t=0 and $t=t^{\prime }$ belong to the “past”, while $t=t^{\prime \prime }$ refers to the “present”, and must be treated differently.

To be able to define a statistical ensemble, one needs the first measurement to yield a non-degenerate eigenvalue $Q_{n_{0}}^{0}$, which prepares the system in the corresponding state $|q_{n_{0}}^{0}\rangle$. The next step consists of evaluating the *probability amplitudes* for all virtual (Feynman) paths, $\left\{q_{n_{2}}^{2}\leftarrow q_{n_{1}}^{1}\leftarrow q_{n_{0}}^{0}\right\}$, starting at $|q_{n_{0}}^{0}\rangle$ and passing through all possible states at $t=t^{\prime }$ and $t=t^{\prime \prime }$ (see Figure 1),

(2) $$A\left(q_{n_{2}}^{2}\leftarrow q_{n_{1}}^{1}\leftarrow q_{n_{0}}^{0}\right)=\langle q_{n_{2}}^{2}|\hat{U}\left(t^{\prime \prime },t^{\prime }\right)|q_{n_{1}}^{1}\rangle \langle q_{n_{1}}^{1}\left|\hat{U}\left(t^{\prime },0\right)\right|q_{n_{0}}^{0}\rangle ,$$

where $\hat{U}\left(t^{\prime \prime },t^{\prime }\right)$ is the system’s evolution operator. An amplitude for obtaining at $t=t^{\prime }$ a value $Q_{m_{1}}^{1}$ is found by summing (2) over all states $|q_{n_{1}}^{1}\rangle$, consistent with $Q_{m_{1}}^{1}$, ${\hat{Q}}^{1}\left|q_{n_{1}}^{1}\rangle =Q_{m_{1}}^{1}\right|q_{n_{1}}^{1}\rangle$,

(3) $$A\left(q_{n_{2}}^{2}\leftarrow Q_{m_{1}}^{1}\leftarrow q_{n_{0}}^{0}\right)=\sum_{n_{1}=1}^{N}\Delta \left(Q_{m_{1}}^{1}-\langle q_{n_{1}}^{1}\left|{\hat{Q}}^{1}\right|q_{n_{1}}^{1}\rangle \right)\times A\left(q_{n_{2}}^{2}\leftarrow q_{n_{1}}^{1}\leftarrow q_{n_{0}}^{0}\right).$$

Finally the *probability* for having a sequence of observed outcomes $\left\{Q_{m_{2}}^{2}\leftarrow Q_{m_{1}}^{1}\leftarrow Q_{n_{0}}^{0}\right\}$ is found by summing absolute squares of the amplitudes (3) over the degeneracies of the last eigenvalue $Q_{m_{2}}^{2}$,

(4) $$P\left(Q_{m_{2}}^{2}\leftarrow Q_{m_{1}}^{1}\leftarrow Q_{n_{0}}^{0}\right)=\sum_{n_{2}=1}^{N}\Delta \left(Q_{m_{2}}^{2}-\langle q_{n_{2}}^{2}\left|{\hat{Q}}^{2}\right|q_{n_{2}}^{2}\rangle \right){\left|A\left(q_{n_{2}}^{2}\leftarrow Q_{m_{1}}^{1}\leftarrow q_{n_{0}}^{0}\right)\right|}^{2}.$$

Two relevant observations can be made here. Firstly, the scheme is explicitly causal in the sense that future observations cannot affect the statistics of the ones already made. In particular, summing (4) over all outcomes $Q_{m_{2}}^{2}$ restores the probabilities for the experiment in which only ${\hat{Q}}^{0}$ and ${\hat{Q}}^{1}$ are measured,

(5) $$P\left(Q_{m_{1}}^{1}\leftarrow Q_{n_{0}}^{0}\right)=\sum_{n_{1}=1}^{N}\Delta \left(Q_{m_{1}}^{1}-\langle q_{n_{1}}^{1}\left|{\hat{Q}}^{1}\right|q_{n_{1}}^{1}\rangle \right)\times {\left|A\left(q_{n_{1}}^{1}\leftarrow q_{n_{0}}^{0}\right)\right|}^{2}=\sum_{m_{2}=1}^{M_{2}}P\left(Q_{m_{2}}^{2}\leftarrow Q_{m_{1}}^{1}\leftarrow Q_{n_{0}}^{0}\right).$$

For someone interested in the statistics of only the first two measurements it does not, therefore, matter what would be measured at $t=t^{\prime \prime }$, or if anything would be measured in the future at all.

Secondly, the scheme treats the “past” (any $t<t^{\prime \prime }$) and the “present” (at $t=t^{\prime \prime }$) differently. If the measured eigenvalue is degenerate, in the “past” one sums the amplitudes, as in Equation (3). At “present”, the probabilities $|A\left(q_{n_{2}}^{2}\leftarrow Q_{m_{1}}^{1}\leftarrow q_{n_{0}}^{0}\right){|}^{2}$ are added, as in Equation (4). The latter rule [8] can be traced to the need to ensure causality in the case an operator commuting with ${\hat{Q}}^{2}$ and having *N* distinct eigenvalues is measured in the immediate future at $t=t^{\prime \prime }+\epsilon$, ϵ→0 [10].

The rules, although formulated for systems in a finite-dimensional Hilbert space are readily generalised to the case where the measured operators have continuous spectra. Note also that they can be used to obtain more compact expressions for the probabilities (5) in terms of the projectors onto the eigen-subspaces of the chosen operators (see, e.g., Section 2.2 of [12]).

## 3. Von Neumann Measurements and the Two-Step Histories

The standard approach to quantum measurements involves coupling the system of interest to another degree of freedom (a probe), and deducing the system’s properties from the probe’s statistics. One choice of a probe is a von Neumann pointer (*p*) [13], a one dimensional free particle of a mass *M* with a coordinate *f* and a momentum λ. The pointer is briefly coupled to the system (*s*) at some $t=t^{\prime }$, so that the full Hamiltonian is given by

(6) $$\hat{H}={\hat{H}}^{s}+{\hat{\lambda }}^{2}/2M+\hat{\lambda }\hat{C}\delta \left(t-t^{\prime }\right),$$

where ${\hat{H}}^{s}$ refers to the system, the operator

(7) $$\hat{C}=\sum_{j=1}^{N}C_{j}\left|c_{j}\rangle \langle c_{j}\right|,$$

represents the system’s variable to be measured, and δ(x) is the Dirac delta. We will start by assuming that all eigenvalues of $\hat{C}$, $C_{j}$, j=1,⋯,N are different, and return to degenerate $C_{j}$’s at the end of the next Section.

A possible experiment consists of preparing the composite *{system*+*pointer}* in a known initial state measuring ${\hat{Q}}^{0}$, applying the coupling (6), and then making a measurement on the pointer, ${\hat{Q}}^{1}$. The purpose of the experiment is to learn something about the system in the absence of the probe. We, therefore, have a two-step history, which can be treated by the method of Section 2. At t=0 the system and the pointer are prepared in states $|b_{i}\rangle$ and |G〉, respectively. In particular, we suppose that the outcome of ${\hat{Q}}^{0}$ is $Q_{1}^{0}$, so

(8) $$\begin{array}{ccc} {\hat{Q}}^{0} & = & {\hat{B}}^{s+p}\equiv |b_{i}\rangle |G\rangle \langle b_{i}\left|\langle G\right|,\;Q_{1}^{0}=1,\;Q_{m_{0}\neq 1}^{0}=0 \end{array}$$

(9) $$\begin{array}{ccc} {\hat{Q}}^{1} & = & {\hat{\chi }}^{p}=\sum_{m=-\cdots ,0,1,\cdots }\chi_{m}\int_{\Delta_{m}}\left|\chi \rangle \langle \chi \right|d\chi ,\;\; \end{array}$$

with $\Delta_{m}\equiv \left[\Delta \left(m-1/2\right),\Delta \left(m+1/2\right)\right]$ and ${\hat{\chi }}^{p}$ acts only on the pointer.

Thus, obtaining an outcome $Q_{1}^{0}=1$ one prepares the composite in an initial state

(10) $$|\Phi_{0}\rangle =|b_{i}\rangle |G\rangle ,\;|G\rangle =\int dfG\left(f\right)|f\rangle .$$

In what follows, we will assume G(f) to be a real symmetric Gaussian,

(11) $$G\left(f\right)={\left(\pi \Delta f^{2}/2\right)}^{-1/4}\exp\left(-f^{2}/\Delta f^{2}\right),$$

where the width Δf determines the uncertainty in the pointer’s *initial position* and, therefore, affects the accuracy of the measurement.

To describe the pointer at $t=t^{\prime }$ we will use a continuous basis |χ〉, $\langle \chi_{1}|\chi_{2}\rangle =\delta \left(\chi_{2}-\chi_{1}\right)$, and the measured operator, ${\hat{\chi }}^{p}$, with a discrete spectrum $Q_{m}^{1}=\chi_{m}$, m=⋯,−1,0,1,⋯. Thus, after observing an outcome $\chi_{m}$ one knows that the (yet undefined) variable χ has a value in an interval Δ(m−1/2)≤χ<Δ(m+1/2). The newly introduced parameter Δ determines the accuracy with which the pointer is *read*. Note that an eigenvalue $\chi_{m}$ is highly degenerate, since ${\hat{\chi }}^{p}\left|\chi \rangle \right|c_{j}\rangle =\chi_{m}\left|\chi \rangle \right|c_{j}\rangle$, for any Δ(m−1/2)≤χ<Δ(m+1/2), and j=1,2,⋯,N.

The evolution operator for the composite {*system* + *pointer*} is a product

(12) $$\hat{U}\left(t^{\prime },0\right)=\exp\left(-i\hat{\lambda }\hat{C}\right){\hat{U}}^{s}\left(t^{\prime },0\right){\hat{U}}^{p}\left(t^{\prime },0\right),$$

where ${\hat{U}}^{s}\left(t^{\prime },0\right)=\exp\left(-i{\hat{H}}^{s}t^{\prime }\right)$, and ${\hat{U}}^{p}\left(t^{\prime },0\right)=\exp\left(-i{\hat{\lambda }}^{2}t^{\prime }/2M\right)$.

Following the recipe of Section 2 we write down the probability amplitudes for all virtual (Feynman) paths $\left\{c_{i},\chi \leftarrow b_{i},G\right\}$ in the composite’s Hilbert space, $H^{p+s}=H^{p}⊗H^{s}$,

(13) $$A\left(c_{i},\chi \leftarrow b_{i},G\right)=\langle \chi |\langle c_{j}|\hat{U}\left(t^{\prime },0\right)\left|b_{i}\rangle \right|G\rangle =G_{j}\left(\chi ,t^{\prime }\right)A^{s}\left(c_{j}\leftarrow b_{i}\right),$$

where

(14) $$A^{s}\left(c_{j}\leftarrow b_{i}\right)=\langle c_{j}\left|{\hat{U}}^{s}\left(t^{\prime },0\right)\right|b_{i}\rangle$$

is the systems transition amplitude [8] between the states $|b_{i}\rangle$ and $|c_{j}\rangle$, defined in the absence of the pointer, and the factor $G_{j}\left(\chi ,t^{\prime }\right)$ can be written as

(15) $$G_{j}\left(\chi ,t^{\prime }\right)=\int \langle f+C_{j}|{\hat{U}}^{p}\left(t^{\prime },0\right)\left|G\rangle \langle \chi \right|f\rangle df,$$

since $\exp\left(-i\hat{\lambda }\hat{C}\right)|c_{j}\rangle |f\rangle =|c_{j}\rangle |f+C_{j}\rangle$.

With only two measurements, all paths $\left\{c_{i},\chi \leftarrow b_{i},G\right\}$ lead to distinguishable (orthogonal) final states, and the probability to have a pointer reading $\chi_{m}$ is found by adding absolute squares of the amplitudes (13)

(16) $$P\left(\chi_{m}\leftarrow b_{i},G\right)=\sum_{j}\int_{\Delta_{m}}d\chi {\left|G_{j}\left(\chi ,t^{\prime }\right)\right|}^{2}{\left|A^{s}\left(c_{j}\leftarrow b_{i}\right)\right|}^{2}.$$

Therefore, regardless of how accurately the meter was prepared and read, all information about the phases of the amplitudes $A^{s}\left(c_{j}\leftarrow b_{i}\right)$ is lost. This is because, according to the rules of Section 2 none of the virtual paths are allowed to interfere. For someone still wishing to determine the system’s initial state $|b_{i}\rangle$, the standard way to proceed is to choose a different operator ${\hat{C}}^{\prime }$, $[{\hat{C}}^{\prime },\hat{C}]\neq 0$ repeat the measurement, and use the obtained data [2,3,4,5,6,7]. We will, however, consider a different problem, in order to exploit the interference associated with the measurements made in the “past”.

## 4. From Probabilities to Amplitudes. Three-Step Histories

Suppose next that an additional measurement is made on the system at a $t^{\prime \prime }>t^{\prime }$. Now the measurement made on the pointer at $t=t^{\prime }$ belongs to the past, and a different rule will apply.

The new experiment is as follows. At t=0 the system and the pointer are prepared in a state $|b_{i}\rangle |G\rangle$, and coupled according to (6) just before $t=t^{\prime }$. At $t=t^{\prime }$, a measurement made on the pointer yields an outcome $\chi_{m}$. At $t=t^{\prime \prime }$ the outcome $\chi_{m}$ is recorded, but only if a measurement ${\hat{D}}^{s}$ made on the system at $t=t^{\prime \prime }$ yields a particular outcome $D_{k}$. The three steps are repeated enough times to evaluate the probabilities of having an outcome $\chi_{m}$, given a later outcome $D_{k}$. The purpose of the experiment is to recover the values of the system’s amplitudes, defined in the absence of the pointer.

This is a three-step measurement, for which we have

(17) $$\begin{array}{cc} {\hat{Q}}^{0} & ={\hat{B}}^{s+p}\equiv |b_{i}\rangle |G\rangle \langle b_{i}\left|\langle G\right|,\;Q_{1}^{0}=1,\;Q_{m_{0}\neq 1}^{0}=0, \end{array}$$

(18) $$\begin{array}{cc} {\hat{Q}}^{1} & ={\hat{\chi }}^{p}=\sum_{m=-\cdots ,1,0,1,\cdots }\chi_{m}\int_{\Delta_{m}}\left|\chi \rangle \langle \chi \right|d\chi , \end{array}$$

(19) $$\begin{array}{cc} {\hat{Q}}^{2} & ={\hat{D}}^{s}=\sum_{k=1}^{N}D_{k}\left|d_{k}\rangle \langle d_{k}\right|. \end{array}$$

We will assume the eigenvalues $D_{k}$ to be non-degenerate provided ${\hat{D}}^{s}$ is acting in the Hilbert space $H^{s}$ of the system. They are, however, highly degenerate, if ${\hat{D}}^{s}$ acts the $H^{s+p}$, since ${\hat{D}}^{s}|d_{k}\rangle |\chi \rangle =D_{k}\left|d_{k}\rangle \right|\chi \rangle$, for any −∞<χ<∞.

Next we apply the rules of Section 2. Evaluating the amplitudes for all virtual paths in $H^{s+p}$, connecting $|b_{i}\rangle |G\rangle$ with $\left|\chi \rangle \right|c_{j}\rangle$, and $\left|\chi \rangle \right|c_{j}\rangle$ with $|d_{k}\rangle |\chi^{\prime }\rangle$ [cf. Equation (2)], we find

(20) $$A\left(d_{k},\chi^{\prime }\leftarrow c_{j},\chi \leftarrow b_{i},G\right)\equiv \langle \chi^{\prime }\left|{\hat{U}}^{p}\left(t^{\prime \prime }-t^{\prime }\right)\right|\chi \rangle G_{j}\left(\chi ,t^{\prime }\right)A^{s}\left(d_{k}\leftarrow c_{j}\leftarrow b_{i}\right)$$

where

(21) $$A^{s}\left(d_{k}\leftarrow c_{j}\leftarrow b_{i}\right)=\langle d_{k}|{\hat{U}}^{s}\left(t^{\prime \prime },t^{\prime }\right)|c_{j}\rangle \times \langle c_{j}|{\hat{U}}^{s}\left(t^{\prime }\right)|b_{i}\rangle \equiv \left|A_{j}\right|\exp\left(i\phi_{j}\right),$$

is the amplitude for the system to follow a path $\left\{|d_{k}\rangle \leftarrow |c_{j}\rangle \leftarrow |b_{i}\rangle \right\}$ in $H^{s}$.

Summing the amplitudes over the degeneracies of the operator ${\hat{\chi }}^{p}$ acting in the $H^{s+p}$ [cf. Equation (3)], yields

(22) $$A\left(d_{k},\chi^{\prime }\leftarrow \chi_{m}\leftarrow b_{i},G\right)=\sum_{j=1}^{N}\int_{\Delta_{m}}d\chi A\left(d_{k},\chi^{\prime }\leftarrow c_{j},\chi \leftarrow b_{i},G\right).$$

Finally, summing over the degeneracies of ${\hat{D}}^{s}$ in the $H^{s+p}$ [cf. Equation (4)], we have

(23) $$\begin{array}{ccc} P(D_{k} & \leftarrow \chi_{m}\leftarrow b_{i},G)=\int d\chi^{\prime }{\left|A\left(d_{k},\chi^{\prime }\leftarrow \chi_{m}\leftarrow b_{i},G\right)\right|}^{2}\; & \\ \; & =\sum_{jj^{\prime }=1}^{N}I_{jj^{\prime }}\left(m\right)|A_{j^{\prime }}\left|\right|A_{j}|\exp\left[i\left(\phi_{j}-\phi_{j^{\prime }}\right)\right]\equiv \int_{\Delta_{m}}d\chi \rho \left(\chi ,d_{k}\right), \end{array}$$

where $I_{jj^{\prime }}\left(m\right)$ is the overlap matrix of the pointer’s states,

(24) $$I_{jj^{\prime }}\left(m\right)\equiv \int_{\Delta_{m}}d\chi G_{j^{\prime }}^{*}\left(\chi ,t^{\prime }\right)G_{j}\left(\chi ,t^{\prime }\right)=I_{j^{\prime }j}^{*}\left(m\right),$$

and $\rho (\chi ,d_{k})$ is the probability density of the pointer’s readings, obtained for a system ending up in $|d_{k}\rangle$ at $t=t^{\prime \prime }$ (see Appendix A).

The measured system contributes to $P(D_{k}\leftarrow \chi_{m}\leftarrow b_{i},G)$ with the path amplitudes given in (21), whose values can, in principle, be determined by rewriting Equation (23) in an equivalent form,

(25) ∑i=1NIiiXii+2∑j′<j=1NRe[Ijj′]Xjj′−Im[Ijj′]Yjj′=P(Dk←χm←bi,G),

where

(26) $$\begin{array}{cc} X_{ij} & \equiv |A_{i}\left|\right|A_{j}|\cos\left(\phi_{i}-\phi_{j}\right),\\ Y_{ij} & \equiv |A_{i}\left|\right|A_{j}|\sin\left(\phi_{i}-\phi_{j}\right). \end{array}$$

The system of linear equations (25) can be solved if the probabilities $P(D_{k}\leftarrow \chi_{m}\leftarrow b_{i},G)$ have been measured for $N^{2}$ different intervals $\Delta_{m}$, $m=1,\cdots ,N^{2}$. Solving Equation (26) for $|A_{i}|$ and $\phi_{i}$, i=1,⋯,N, one will be able to determine the values of all amplitudes $A^{s}\left(d_{k}\leftarrow c_{j}\leftarrow b_{i}\right)\equiv \left|A_{j}\right|\exp\left(i\phi_{j}\right)$ up to an unimportant overall phase.

To conclude the Section, we note that the measured operator $\hat{C}$ may have J<N degenerate eigenvalues,

(27) $$\hat{C}=\sum_{j=1}^{J}C_{j}\sum_{n=1}^{N}\Delta \left(C_{j}-\langle c_{n}\left|\hat{C}\right|c_{n}\rangle \right)\left|c_{n}\rangle \langle c_{n}\right|\equiv \sum_{j=1}^{J}C_{j}{\hat{\pi }}_{j},$$

where ${\hat{\pi }}_{j}$ projects onto its *j*-th eigen-subspace. In this case the analysis remains the same, except that *N* is replaced by *J*, and the *J* amplitudes to be determined,

(28) $$A^{s}\left(d_{k}\leftarrow C_{j}\leftarrow b_{i}\right)=\sum_{n=1}^{N}\Delta \left(C_{j}-\langle c_{n}\left|\hat{C}\right|c_{n}\rangle \right)\times \langle d_{k}\left|{\hat{U}}^{s}\left(t^{\prime \prime },t^{\prime }\right){\hat{\pi }}_{j}{\hat{U}}^{s}\left(t^{\prime },0\right)\right|b_{i}\rangle ,$$

result from the interference between the virtual paths (21) not distinguished by a measurement of $\hat{C}$.

Next we see how the scheme would work in the simplest case of a two-level system, N=2.

## 5. An Inverse Measurement Problem

We can write Equation (23) as

(29) $$\langle Z|\tilde{I}\left(m_{\mu }\right)|Z\rangle =P\left(D_{k}\leftarrow \chi_{m_{\mu }}\leftarrow b_{i},G\right),\;m_{\mu }=1,\cdots ,2N,$$

where |Z〉 is a complex “vector” with the components $Z_{j}\equiv A^{s}\left(d_{k}\leftarrow c_{j}\leftarrow b_{i}\right)$, j=1,⋯,N, and $\tilde{I}\left(m_{\mu }\right)$ is an “operator” with matrix elements $I_{jj^{\prime }}\left(m_{\mu }\right)$, $j,j^{\prime }=1,2,\cdots ,N$, and the subindex μ accounts for the specific subset of intervals used. The problem now takes a more familiar form. One needs to find the components of a (fictitious) state |Z〉, given the expectation values of the hermitian operators $\tilde{I}\left(m_{\mu }\right)$, $m_{\mu }=1,2,\cdots ,2N$.

Equationation (29) is particularly useful in the case N=2, where |Z〉 can be seen as an unnormalised state of a fictitious “spin”, and $\tilde{I}\left(m\right)$ can be expanded in terms of the Pauli matrices (${\hat{\sigma }}_{1}=\hat{I}$, ${\hat{\sigma }}_{2}={\hat{\sigma }}_{x}$, ${\hat{\sigma }}_{3}={\hat{\sigma }}_{y}$, and ${\hat{\sigma }}_{4}={\hat{\sigma }}_{z}$),

(30) $$\tilde{I}\left(m_{\mu }\right)=\sum_{\nu =1}^{4}\beta_{\mu \nu }{\hat{\sigma }}_{\nu },\;\mu =1,\cdots ,4,$$

with four intervals $\Delta_{m_{\mu }}$ corresponding to the pointer’s readings $\chi_{m_{\mu }}$, chosen at one’s convenience. The resulting four equations (29) determine the “spin”’s projections $\langle X|{\hat{\sigma }}_{x,y,z}|X\rangle$ onto the three spatial axes, as well as the state’s norm, 〈X|X〉,

(31) $$\sum_{\nu =1}^{4}\beta_{\mu \nu }\langle Z\left|{\hat{\sigma }}_{\nu }\right|Z\rangle =P\left(D_{k}\leftarrow \chi_{m_{\mu }}\leftarrow b_{i},G\right),\;\mu =1,\cdots ,4.$$

The polar angles θ and ϕ of the axis along which the “spin” is polarised,

(32) $$\begin{array}{cc} \sin\theta \cos\phi & =\langle Z|{\hat{\sigma }}_{x}\left|Z\rangle /\langle Z\right|Z\rangle ,\\ \sin\theta \sin\phi & =\langle Z|{\hat{\sigma }}_{y}\left|Z\rangle /\langle Z\right|Z\rangle ,\\ \cos\theta & =\langle Z|{\hat{\sigma }}_{z}\left|Z\rangle /\langle Z\right|Z\rangle , \end{array}$$

determine the “spin”’s state ${\left[\cos\left(\theta /2\right),\exp\left(i\phi \right)\sin\left(\theta /2\right)\right]}^{T}$ up to an arbitrary overall phase and, returning to our original notations, we have the desired result,

(33) $$\begin{array}{cc} A^{s}\left(d_{k}\leftarrow c_{1}\leftarrow b_{i}\right) & =\sqrt{\langle X|X\rangle }\cos\left(\theta /2\right),\\ A^{s}\left(d_{k}\leftarrow c_{2}\leftarrow b_{i}\right) & =\sqrt{\langle X|X\rangle }\exp\left(i\phi \right)\sin\left(\theta /2\right). \end{array}$$

Next we see how the scheme will work in practice.

## 6. Double-Slit Interference

**Figure 2:**
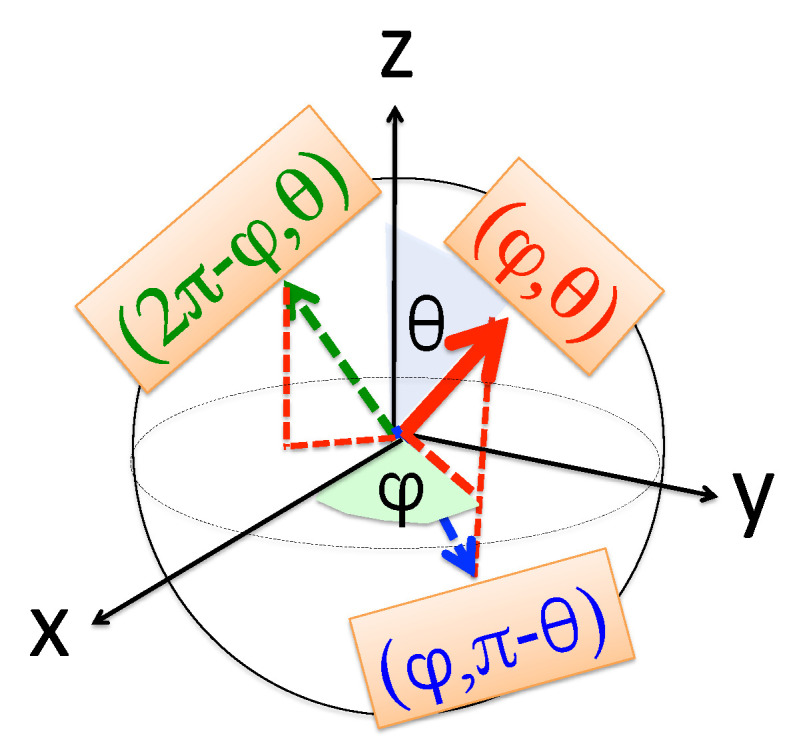
With only two amplitudes, $A^{s}\left(d_{k}\leftarrow c_{1}\leftarrow b_{i}\right)$ and $A^{s}\left(d_{k}\leftarrow c_{2}\leftarrow b_{i}\right)$ to be evaluated, the “spin”’s state |Z〉 in Equation (31) lies on a Bloch sphere. If the pointer has no own dynamics, e.g., M→∞, evaluating only its position (momentum) distribution leaves the azimuthal angle ϕ (polar angle θ) indeterminate. The problem is remedied if the pointer’s state is allowed to spread, as discussed in Section 7.

It is natural to start with the simplest case, where one measures the final position of a massive pointer, |χ〉=|f〉, and

(34) $$M\to \infty ,\;\mathrm{so}\;\mathrm{that}\;{\hat{U}}^{p}\left(t^{\prime }-t\right)\to 1,$$

which moves only when it interacts with the system, and whose state remains the same once this interaction is over [13]. If so, the matrices $\tilde{I}\left(m\right)$ are real,

(35) $$I_{jj^{\prime }}^{pos}\left(m\right)=\int_{\Delta_{m}}dfG\left(f-C_{1}\right)G\left(f-C_{2}\right),$$

and the coefficient multiplying ${\hat{\sigma }}_{y}$ in Equation (30) vanishes, $\beta_{\mu 3}\equiv 0$. One can still solve any three of Equation (31) for 〈Z|Z〉, $\langle Z|{\hat{\sigma }}_{x}|Z\rangle$, and $\langle Z|{\hat{\sigma }}_{z}\left|Z\rangle /\langle Z\right|Z\rangle$ but would be unable to decide between ϕ and 2π−ϕ, as illustrated in Figure 2. (Note that the problem is exacerbated for N>2 where N−1 signs would remain indeterminate when calculating the relative phases.)

Measuring instead the final pointer’s momentum |χ〉=|λ〉, ${\langle \lambda |f\rangle =\left(2\pi \right)}^{-1/2}\exp\left(-i\lambda f\right)$, one encounters a similar difficulty. In this case we have

(36) $$\begin{array}{cc} I_{jj^{\prime }}^{mom}\left(m\right) & =\int_{\Delta_{m}}d\lambda {\left|\tilde{G}\left(\lambda \right)\right|}^{2}\exp\left[i\left(C_{j}-C_{j^{\prime }}\right)\lambda \right],\\ \tilde{G}\left(\lambda \right) & ={\left(2\pi \right)}^{-1/2}\int df\exp\left(-i\lambda f\right)G\left(f\right), \end{array}$$

so that $\beta_{\mu 4}\equiv 0$, and having solved the three remaining equations one will not be able to decide between θ and π−θ (see Figure 2).

However, provided both $P(D_{k}\leftarrow f_{m}\leftarrow b_{i},G)$ and $P(D_{k}\leftarrow \lambda_{m}\leftarrow b_{i},G)$ have been measured independently in two different experiments, one can combine the results to obtain the four Equation (31). For example, choosing any three equations employing $I_{jj^{\prime }}^{pos}\left(m\right)$ and $P(D_{k}\leftarrow f_{m}\leftarrow b_{i},G)$, and one using $I_{jj^{\prime }}^{mom}\left(m\right)$ and $P(D_{k}\leftarrow \lambda_{m}\leftarrow b_{i},G)$, will determine the two amplitudes (33) unambiguously (up to a global phase).

Finally we note that the case of a two-level system is conceptually similar to a Young’s double-slit experiment. Here the two states $|c_{1}\rangle$ and $|c_{2}\rangle$ play the roles of the two holes, and the target state $|d_{k}\rangle$, together with its orthogonal companion, $|d_{k^{\prime }}\rangle$, $\langle d_{k}|d_{k^{\prime }}\rangle =0$ are the “points on the screen”. Unperturbed probabilities $|A^{s}\left(d_{k}\leftarrow c_{1}\leftarrow b_{i}\right)+A^{s}\left(d_{k}\leftarrow c_{2}\leftarrow b_{i}\right){|}^{2}$ and $|A^{s}\left(d_{k^{\prime }}\leftarrow c_{1}\leftarrow b_{i}\right)+A^{s}\left(d_{k^{\prime }}\leftarrow c_{2}\leftarrow b_{i}\right){|}^{2}$ correspond to having an “interference pattern on the screen”. Thus, if a von Neumann measurement perturbs the interference pattern (the probability to be detected in $|d_{k}\rangle$ is $\int \rho \left(f,d_{k}\right)df=\int \rho \left(\lambda ,d_{k}\right)d\lambda \neq {\left|A^{s}\left(d_{k}\leftarrow c_{1}\leftarrow b_{i}\right)+A^{s}\left(d_{k}\leftarrow c_{2}\leftarrow b_{i}\right)\right|}^{2}$), yet does not destroy it completely, and the values of the path amplitudes $A^{s}\left(d_{k^{\prime }}\leftarrow c_{j}\leftarrow b_{i}\right)$ can be deduced from the measurement’s statistics.

## 7. A Simple Example

To see the efficiency of the proposed scheme, we first change it a little. It is possible to avoid mixing the results of measuring the pointer’s position and momentum, if the condition (34) is relaxed, and the pointer’s initial state |G〉 is allowed to spread in the coordinate space. Choosing |χ〉=|f〉, from (15) we have

(37) $$\begin{array}{ccc} G_{j}\left(\chi ,t^{\prime }\right) & = & \frac{2^{1/4}\Delta f^{1/4}}{\pi^{1/4}\Delta f_{t^{\prime }}}\exp\left[\frac{{\left(f-C_{j}\right)}^{2}}{\Delta f_{t^{\prime }}^{2}}\right],\\ \Delta f_{t^{\prime }} & \equiv & {\left(\Delta f^{2}+2it^{\prime }/M\right)}^{1/2}. \end{array}$$

The appearance of a “complex width” $\Delta f_{t^{\prime }}$ allows one to determine all coefficients $\beta_{\mu \nu }$ in Equation (30) from the statistics of the final pointer’s positions. Explicitly, from (24) we have

(38) βμ1=[I11(mμ)+I22(mμ)]/2,βμ2=Re[I12(mμ)],βμ3=−Im[I12(mμ)],βμ4=[I11(mμ)−I22(mμ)]/2.

**Figure 3:**
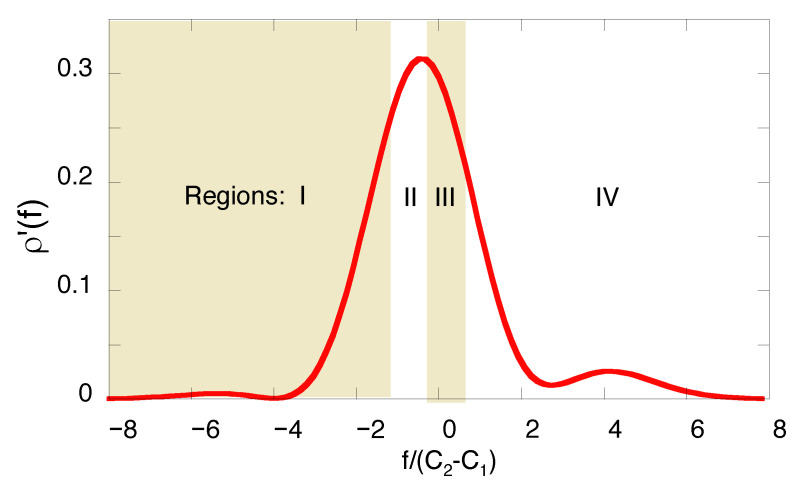
Normalised probability density, $\rho^{\prime }\left(f\right)=\rho \left(f\right)/\int \rho \left(f^{\prime }\right)df^{\prime }$, of the pointer’s readings for a two-level system making a transition between the initial and final states (41). Dividing the range of *f* into four equally probable regions ensures the same accuracy in approximating the probabilities by the relative frequencies in the r.h.s. of Equation (39).

In an actual experiment set-up to evaluate the amplitudes $A^{s}\left(d_{k^{\prime }}\leftarrow c_{j}\leftarrow b_{i}\right)$ a successful post-selection of the system in the final state, $|d_{k}\rangle$, will occur *K* times out of the total number of trials, $K^{total}$. It is convenient to divide the full range of *f* into four regions, $\Delta_{\mu }$, μ=I,II,III,IV, each containing a quarter of all cases, K(μ)=K/4 (see Figure 3). To simulate the measurements, we use a random number generator, obtain four numbers K(I), K(II), K(III), and K(IV), $\sum_{\mu =I}^{IV}K\left(\mu \right)=K$, replace the probabilities in the r.h.s. of Equation (31) by their estimates,

(39) $$\sum_{\nu =1}^{4}\beta_{\mu \nu }\langle Z\left|{\hat{\sigma }}_{\nu }\right|Z\rangle =\frac{K(\mu )}{K^{total}}\overset{K^{total}\to \infty }{\to }\frac{K}{4K_{total}},\;\mu =I,\cdots ,IV,$$

and solve Equation (39) for different values of $K^{total}$.

**Figure 4:**
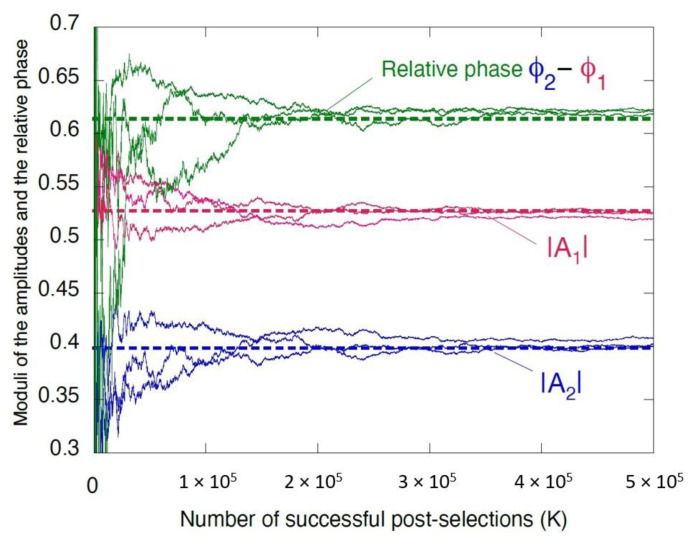
The moduli and relative phase of the amplitudes $A^{s}\left(d_{k}\leftarrow c_{j}\leftarrow b_{i}\right)\equiv \left|A_{j}\right|\exp\left(i\phi_{j}\right)$, j=1,2, as function of the number of the successful post-selection, *K*, evaluated as *K* increases by 100. The dashed lines indicate the exact values.

The results of three simulations for

(40) $${\hat{H}}^{s}=\omega {\hat{\sigma }}_{z},\;\hat{C}=\frac{1}{2}\sigma_{z}=\left[\right|c_{1}\rangle \langle c_{1}\left|-\right|c_{2}\rangle \langle c_{2}\left|\right]/2,\;C_{2}=-C_{1}=1/2,$$

arbitrarily chosen (unnormalised) initial and final states,

(41) $$\begin{array}{cc} |b_{i}\rangle & =\left(2.5+4i\right)|c_{1}\rangle +\left(6+3i\right)|c_{2}\rangle ,\\ |d_{k}\rangle & =\left(3+4i\right)|c_{1}\rangle +\left(5+7i\right)|c_{2}\rangle , \end{array}$$

and

(42) $$\omega t^{\prime }=\pi /3,\;t^{\prime }/M=\pi /6,\;\Delta f/\left(C_{2}-C_{1}\right)=0.5,$$

are shown in Figure 4. It takes approximately $K∼10^{5}-10^{6}$ successful post-selections in order to recover the amplitudes of $A^{s}\left(d_{k^{\prime }}\leftarrow c_{j}\leftarrow b_{i}\right)$, given the values in Equation (42). Next we discuss the two limiting cases, in which the method of this Section will fail.

## 8. Accurate (Strong) and Inaccurate (Weak) Limits

**Figure 5:**
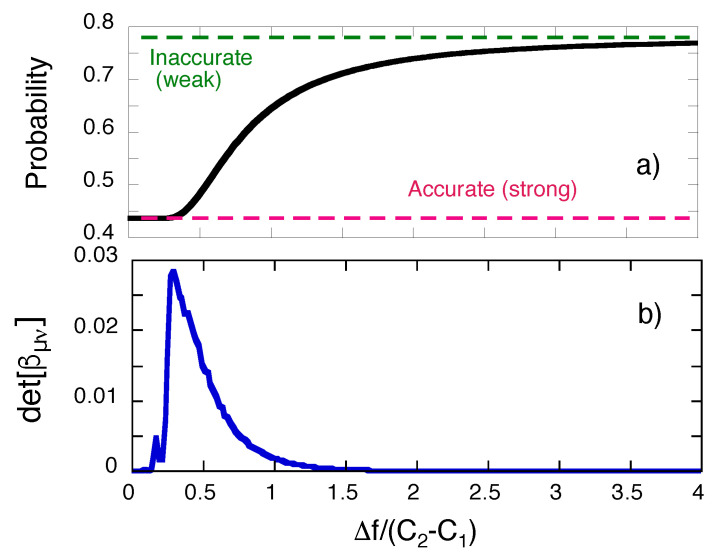
(**a**) The probability $P(D_{k}\leftarrow b_{i},G)$ in Equation (43) as function of the uncertainty in the pointer’s initial position, Δf. (**b**) the determinant of the matrix $\beta_{\mu \nu }$ in Equation (39) vs. Δf.

The method of Section 8 fails, or at least becomes impractical as Δf→0 or Δf→∞. The uncertainty in the initial pointer’s position determines the perturbation suffered by the measured system, as shown in Figure 5a, where the probability of detecting the system in the final state $|d_{k}\rangle$,

(43) $$P\left(D_{k}\leftarrow b_{i},G\right)=\sum_{m=I,IV}P\left(D_{k}\leftarrow f_{m}\leftarrow b_{i},G\right)=\int_{-\infty }^{\infty }\rho \left(f,d_{k}\right)df,$$

is seen to vary from $|A^{s}\left(d_{k}\leftarrow c_{1}\leftarrow b_{i}\right){|}^{2}+{\left|A^{s}\left(d_{k}\leftarrow c_{2}\leftarrow b_{i}\right)\right|}^{2}$ to $|A^{s}\left(d_{k}\leftarrow c_{1}\leftarrow b_{i}\right)+A^{s}\left(d_{k}\leftarrow c_{2}\leftarrow b_{i}\right){|}^{2}$, as Δf increases from 0 to ∞. In both limits the matrix in the l.h.s. of Equation (39) becomes singular, as shown in Figure 5b.

In particular, if the initial position of the pointer is highly uncertain, Δf→∞, from (23) we have

(44) $$\int_{-\infty }^{\infty }G_{j^{\prime }}^{*}\left(f,t^{\prime }\right)G_{j}\left(f,t^{\prime }\right)df=\int_{-\infty }^{\infty }G\left(f-C_{j^{\prime }}\right)G\left(f-C_{j}\right)df\overset{\Delta f\to \infty }{\to }1.$$

The pointer decouples from the system, and the interference between the two virtual paths is preserved,

(45) $$P\left(D_{k}\leftarrow b_{i},G\right)\to {\left|A^{s}\left(d_{k}\leftarrow c_{1}\leftarrow b_{i}\right)+A^{s}\left(d_{k}\leftarrow c_{2}\leftarrow b_{i}\right)\right|}^{2}.$$

Note that the same effect can be achieved by leaving the width Δf constant, and multiplying the coupling term in Equation (6), ${\hat{H}}_{int}\equiv \hat{\lambda }\hat{C}=-i\partial_{f}\hat{C}$, by a constant α→0. Indeed, scaling the pointer’s coordinate by choosing $f^{\prime }=\alpha f$, would result in

(46) $$G\left(f\right)\to G\left(f^{\prime }/\alpha \right),\;\mathrm{and}\;{\hat{H}}_{int}\left(f\right)\to \alpha {\hat{H}}_{int}\left(f^{\prime }\right).$$

If, on the other hand, the initial position of the pointer is known accurately, Δf→0, Equation (23) yields

(47) $$P\left(D_{k}\leftarrow b_{i},G\right)\to |A^{s}\left(d_{k}\leftarrow c_{1}\leftarrow b_{i}\right){|}^{2}+{\left|A^{s}\left(d_{k}\leftarrow c_{2}\leftarrow b_{i}\right)\right|}^{2},$$

since by (15)

(48) $$\int_{-\infty }^{\infty }G_{j^{\prime }}^{*}\left(f,t^{\prime }\right)G_{j}\left(f,t^{\prime }\right)df\overset{\Delta f\to 0}{\to }\delta_{jj^{\prime }}.$$

Thus, an accurately set pointer strongly perturbs the system by completely destroying interference between the virtual paths, even when the spreading of its initial state is taken into account.

## 9. Averages and the “Weak Measurements”

Another possibility to explore the limits Δf→0 and Δf→∞ is to evaluate the moments of the distribution of the pointer’s readings (23),

(49) $$\langle \chi^{l}\rangle \equiv \int_{-\infty }^{\infty }\chi^{l}\rho \left(\chi ,d_{k}\right)d\chi /\int_{-\infty }^{\infty }\rho \left(\chi ,d_{k}\right)d\chi ,\;l=0,1,2,\cdots$$

These are, of course, also expressed in terms of the system’s path amplitudes $A^{s}\left(d_{k}\leftarrow c_{1}\leftarrow b_{i}\right)\equiv \left|A_{j}\right|\exp\left(i\phi_{j}\right)$, and we will look at the ρ’s first moments in the “strong” and the “weak” limits. For simplicity, we will restore the condition (34) or, what is the same, assume that the times $t^{\prime }$ and $t^{\prime \prime }-t^{\prime }$ are so short that ${\hat{U}}^{p}\left(t^{\prime }\right),{\hat{U}}^{p}\left(t^{\prime \prime }-t^{\prime }\right)\approx 1$.

Expansions around Δf→0 are not particularly interesting. Bearing in mind that $\int fG^{2}\left(f-C_{j}\right)df=C_{j}$, in the two-level case of Section 6, for |χ〉=|f〉 we have

(50) $$\langle f\rangle \approx \langle f\rangle_{\Delta f=0}-F_{0}\left(\Delta f,C_{j}\right)\frac{|A_{1}\left|\right|A_{2}|\cos\left(\phi_{1}-\phi_{2}\right)}{|A_{1}{|}^{2}+{\left|A_{2}\right|}^{2}}+F_{1}\left(\Delta f,C_{j}\right)\frac{|A_{1}\left|\right|A_{2}|\cos\left(\phi_{1}-\phi_{2}\right)}{\left(\right|A_{1}{|}^{2}+|A_{2}{{|}^{2})}^{2}},$$

where $\langle f\rangle_{\Delta f=0}=(C_{1}|A_{1}{|}^{2}+C_{2}|A_{2}{|}^{2})/(|A_{1}{|}^{2}+|A_{2}{|}^{2})$ is the average, obtained in a highly accurate measurement, and the factors $F_{0}$ and $F_{1}$, which only depend on the parameters of the pointer and the eigenvalues $C_{j}$, rapidly decrease for Δf→0 (See Appendix B).

Similarly, since $\int p|\tilde{G}{\left(p\right)|}^{2}dp=0$, for the mean pointer’s momentum, |χ〉=|λ〉 we obtain

(51) $$\langle \lambda \rangle \approx L\left(\Delta f,C_{j}\right)\frac{|A_{1}\left|\right|A_{2}|\sin\left(\phi_{1}-\phi_{2}\right)}{|A_{1}{|}^{2}+{\left|A_{2}\right|}^{2}},$$

where $L\left(\Delta f,C_{j}\right)\overset{\Delta f\to 0}{\to }0$ is given in the Appendix B. Thus, some information about the relative phase of the two path amplitudes can be obtained from accurate yet not too accurate measurements. However, the expressions (50) and (51) are cumbersome and, as we already said, not particularly interesting.

The opposite limit Δ→∞ is involved in the controversy surrounding the so-called “weak measurements”. Returning to the notations of Section 3 and noting that $\int dffG\left(f-C_{1}\right)G\left(f-C_{2}\right)\approx \int dff\left[G\left(f\right)-\partial_{f}G\left(f\right)C_{1}\right]\left[G\left(f\right)-\partial_{f}G\left(f\right)C_{2}\right]\approx \left(C_{1}+C_{2}\right)/2$, for the pointer’s mean position we have

(52) 〈f〉≈〈Z|(1^C1+C22+σ^xC1+C22+σ^zC1−C22|Z〉〈Z|1^+σ^x|Z〉=ReC1As(dk←c1←bi)+C2As(dk←c2←bi)As(dk←c1←bi)+As(dk←c2←bi).

Similarly, for the mean pointer’s momentum we find

(53) 〈λ〉→Δf→∞Var(λ)〈Z|σ^x(C1−C2)|Z〉〈Z|1^+σ^x|Z〉=Var(λ)×ImC1As(dk←c1←bi)+C2As(dk←c2←bi)As(dk←c1←bi)+As(dk←c2←bi)

where Var(λ) is the variance of the distribution of the momenta in the initial pointer’s state |G〉,

(54) $$\mathrm{Var}\left(\lambda \right)\equiv \int d\lambda \lambda^{2}\tilde{G}{\left(\lambda \right)}^{2}/\int d\lambda \tilde{G}{\left(\lambda \right)}^{2}\overset{\Delta f\to \infty }{\to }0,$$

which vanishes when G(f) becomes very broad in the coordinate space, and $\tilde{G}{\left(\lambda \right)}^{2}\to \delta \left(\lambda \right)$.

Equationations (50)–(53), although different, illustrate the same point. Any average, evaluated for a pointer coupled, as in Equation (6), to a system making a transition between initial and final states will have to be expressed in terms of certain combinations of the system’s transition amplitudes, defined in the absence of the pointer. Transition amplitudes are the basic elements of the description of quantum motion [8], and this is really all that can be said about this matter.

The above analysis relates to the so called “weak values” WV(for a recent review see [9]). For short enough $t^{\prime }$ and $t^{\prime \prime }-t^{\prime }$, ${\hat{U}}^{s}\left(t^{\prime }\right),{\hat{U}}^{s}\left(t^{\prime \prime }-t^{\prime }\right)\approx \hat{1}$, the quantity in the square brackets in Equations (52) and (53) reduces to a ratio of matrix elements

(55) $$\frac{C_{1}A^{s}\left(d_{k}\leftarrow c_{1}\leftarrow b_{i}\right)+C_{2}A^{s}\left(d_{k}\leftarrow c_{2}\leftarrow b_{i}\right)}{A^{s}\left(d_{k}\leftarrow c_{1}\leftarrow b_{i}\right)+A^{s}\left(d_{k}\leftarrow c_{2}\leftarrow b_{i}\right)}=\frac{\sum_{j=1,2}\langle d_{k}\left|c_{j}\rangle C_{j}\langle c_{j}\right|b_{i}\rangle }{\sum_{j=1,2}\langle d_{k}\left|c_{j}\rangle j\langle c_{j}\right|b_{i}\rangle }=\frac{\langle d_{k}\left|\hat{C}\right|b_{i}\rangle }{\langle d_{k}|b_{i}\rangle }.$$

Presented in this manner in [14], the r.h.s of Equation (55) was dubbed “the weak value of $\hat{C}$ for a system pre- and post-selected in its initial and final states”, which be can obtained in a particular kind of “weak quantum measurements”. Various weak values have been measured experimentally, yet their place and status within conventional quantum mechanics remain unclear. We have long advocated the interpretation of the “weak measurements” in terms of Feynman’s transition amplitudes, and refer the reader to [15,16,17,18] for an analysis of the role of the Uncertainty Principle and the significance of “anomalous weak values”. Here we further support this view by placing the “weak measurements” within a broader context of measuring the transition amplitudes, absent in the simple two-step histories of Section 2.

## 10. Prediction and Retrodiction

Having recovered the transition amplitudes $A^{s}\left(d_{k^{\prime }}\leftarrow c_{j}\leftarrow b_{i}\right)$, it is reasonable to question the usefulness of what has been found. If the amplitudes are known for all $|c_{j}\rangle$, j=1,⋯,N, they can be used to predict the results of other measurements made on the same system staring in the same $|b_{i}\rangle$, and ending in the same $|d_{k}\rangle$, provided the new operator ${\hat{C}}^{\prime }$ commutes with $\hat{C}$, $[\hat{C},{\hat{C}}^{\prime }]=0$. Indeed, their values is all that required to compute the probabilities $P(D_{k}\leftarrow \chi_{m}\leftarrow b_{i},G)$ in Equation (23), for any choice of G(f), |χ〉, $C_{j}^{\prime }$ and Δ, even if the Hamiltonian of the system, ${\hat{H}}^{s}$, is not known. The task is not entirely trivial for N>2, where ${\hat{C}}^{\prime }$ can have degenerate eigenvalues, and the corresponding amplitudes must be added, as described in Section 2.

On the other hand, little can be learnt about an intermediate measurement of a ${\hat{C}}^{\prime }$ which does not commute with the $\hat{C}$. This is seen already from the N=2 example, discussed in the previous Section. Suppose one replaces $\hat{C}={\hat{\sigma }}_{z}$ with a ${\hat{C}}^{\prime }={\hat{\sigma }}_{x}$, so that now

(56) $$|c_{1}^{\prime }\rangle =\left[\right|c_{1}\rangle +|c_{2}\rangle ]/\sqrt{2},\;|c_{2}^{\prime }\rangle =\left[\right|c_{1}\rangle -\left|c_{2}\rangle \right]/\sqrt{2},$$

and

(57) $$\begin{array}{ccc} A^{s}\left(d_{k}\leftarrow c_{1}^{\prime }\leftarrow b_{i}\right) & = & A^{s}\left(d_{k}\leftarrow c_{1}\leftarrow b_{i}\right)/2+A^{s}\left(d_{k}\leftarrow c_{2}\leftarrow b_{i}\right)/2\\ & + & \langle d_{k}|{\hat{U}}^{s}\left(t^{\prime \prime }-t^{\prime }\right)|c_{1}\rangle \langle c_{2}|{\hat{U}}^{s}\left(t^{\prime }\right)|b_{i}\rangle /2+\langle d_{k}|{\hat{U}}^{s}\left(t^{\prime \prime }-t^{\prime }\right)|c_{2}\rangle \langle c_{1}\left|{\hat{U}}^{s}\left(t^{\prime }\right)\right|b_{i}\rangle /2. \end{array}$$

Of the four quantities in the r.h.s. of Equation (57) needed to evaluate $A^{s}\left(d_{k}\leftarrow c_{1}^{\prime }\leftarrow b_{i}\right)$, only the first two are known from measuring the $\hat{C}$, and this is clearly not enough.

Another use of the path amplitudes (21) and (28) is retrodictive reconstruction of the system’s past. Classically, the knowledge of a system’s current position, velocity, and its Lagrangian is sufficient for predicting its position in the past. Quantally, one may wish to determine the system’s initial state, $|b_{i}\rangle$, from the values of the path amplitudes. The state is fully determined by the coefficients $\langle \varphi_{n}|b_{i}\rangle$ of its expansion in some known basis $|\varphi_{n}\rangle$, $|b_{i}\rangle =\sum_{n=1}^{N}\langle \varphi_{n}\left|b_{i}\rangle \right|\varphi_{n}\rangle$. If the system’s Hamiltonian ${\hat{H}}^{s}$ is known, the operator $\hat{C}$ has non-degenerate eigenvalues [cf. Equation (7)], and the values of $A^{s}\left(d_{k}\leftarrow c_{j}\leftarrow b_{i}\right)$ have been measured, the problem is easily solved. Indeed, using (21) we obtain

(58) $$\begin{array}{cc} \langle \varphi_{n}|b_{i}\rangle & =A^{s}\left(d_{k}\leftarrow c_{n}\leftarrow b_{i}\right)/\langle d_{k}\left|{\hat{U}}^{s}\left(t^{\prime \prime },t^{\prime }\right)\right|c_{n}\rangle ,\\ |\varphi_{n}\rangle & \equiv {\hat{U}}^{s-1}\left(t^{\prime },0\right)|c_{n}\rangle ,\\ \langle \varphi_{n^{\prime }}|\varphi_{n}\rangle & =\langle c_{n^{\prime }}|c_{n}\rangle =\delta_{nn^{\prime }}, \end{array}$$

and with $|b_{i}\rangle$ thus determined, the results of other possible measurements can be predicted.

However, if some of the $\hat{C}$’s eigenvalues are degenerate [cf. Equation (27)], full reconstruction of the initial state is not possible, since important information is lost to interference. From (28) we have

(59) $$A^{s}\left(d_{k}\leftarrow C_{j}\leftarrow b_{i}\right)=\sum_{n=1}^{N}\Delta \left(C_{j}-\langle c_{n}\left|\hat{C}\right|c_{n}\rangle \right)\times \langle d_{k}|{\hat{U}}^{s}\left(t^{\prime \prime },t^{\prime }\right)\left|c_{n}\rangle \langle \varphi_{n}\right|b_{i}\rangle ,$$

so that the values of $\langle \varphi_{n}|b_{i}\rangle$ cannot be recovered from the known values of $A^{s}\left(d_{k}\leftarrow C_{j}\leftarrow b_{i}\right)$.

## 11. Conclusions

In summary, we have shown that the values of system’s transition amplitudes can be deduced from the statistics of an intermediate measurement. The deduction is possible provided the measurement is “fuzzy”, and does not destroy interference between the system’s virtual paths. What distinguishes our method from usual approach to the “Pauli problem” [2,3,4,5,6,7] is its reliance on a type of interference, absent in two-step histories, consisting only of preparation and the actual measurement. With the post-selection step added, the situation is conceptually similar to a double-(multiple-) slit experiment [8], in which a probe, designed to determine the path taken by the system, does its job imperfectly, so a vestige of the interference pattern is retained on the screen. There is a two-way relationship between a result of observation and what can be considered a computational tool, although it is typically more difficult to deduce amplitudes from the probabilities than to construct the probabilities from the known amplitudes.

Finally, it is worth noticing that neither reconstructing the amplitudes as in Section 7, nor evaluating their combinations by measuring the “weak values” of Section 9, would serve to provide a deeper insight into quantum mechanical formalism. If asked “what has been evaluated?” one can only answer “amplitudes”. Moreover, if asked further “what are these amplitudes?” one can only reply “something quantum theory uses to predict the observable probabilities”.

## Appendix A. Derivation of Equation (23)

By definition,

(A1) $$P\left(D_{k}\leftarrow \chi_{m}\leftarrow b_{i},G\right)=\int_{-\infty }^{\infty }d\chi^{\prime }\times |\sum_{j=1}^{N}\int_{\Delta_{m}}d\chi \langle \chi^{\prime }|{\hat{U}}^{p}\left(t^{\prime \prime }-t^{\prime }\right){\left|\chi \rangle G_{j}\left(\chi ,t^{\prime }\right)A^{s}\left(d_{k}\leftarrow c_{j}\leftarrow b_{i}\right)\right|}^{2}.$$

But

(A2) $$\int_{-\infty }^{\infty }d\chi^{\prime }\langle \chi^{\prime \prime }|{\hat{U}}^{p†}\left(t^{\prime \prime }-t^{\prime }\right)|\chi^{\prime }\rangle \langle \chi^{\prime }\left|{\hat{U}}^{p}\left(t^{\prime \prime }-t^{\prime }\right)\right|\chi \rangle =\delta \left(\chi^{\prime \prime }-\chi \right)$$

so that [$\theta_{\Delta_{m}}\left(\chi \right)=1$ inside $\Delta_{m}$, and 0 otherwise]

(A3) $$\int_{\Delta_{m}}d\chi^{\prime \prime }d\chi \delta \left(\chi^{\prime \prime }-\chi \right)G_{j^{\prime }}^{*}\left(\chi^{\prime \prime },t^{\prime }\right)G_{j}\left(\chi ,t^{\prime }\right)=\int_{\Delta_{m}}d\chi \theta_{\Delta_{m}}\left(\chi \right)G_{j^{\prime }}^{*}\left(\chi ,t^{\prime }\right)G_{j}\left(\chi ,t^{\prime }\right)=\int_{\Delta_{m}}d\chi G_{j^{\prime }}^{*}\left(\chi ,t^{\prime }\right)G_{j}\left(\chi ,t^{\prime }\right).$$

Defining

(A4) $$\begin{array}{ccc} \rho (\chi ,d_{k}) & \equiv & \sum_{j}\sum_{j^{\prime }}G_{j^{\prime }}^{*}\left(\chi ,t^{\prime }\right)G_{j}\left(\chi ,t^{\prime }\right)\times A^{s*}\left(d_{k}\leftarrow c_{j^{\prime }}\leftarrow b_{i}\right)A^{s}\left(d_{k}\leftarrow c_{j}\leftarrow b_{i}\right)\\ & = & |\sum_{j}G_{j}\left(\chi ,t^{\prime }\right)A^{s}\left(d_{k}\leftarrow c_{j}\leftarrow b_{i}\right){|}^{2}, \end{array}$$

we obtain Equation (23).

## Appendix B. The Factors ℱ_0_, ℱ_1_, and ℒ in Equations (50) and (51)

In Section 9 we defined

(A5) $$\begin{array}{cc} F_{0}\left(\Delta f,C_{j}\right) & \equiv 2\int G\left(f-C_{1}\right)G\left(f-C_{2}\right)df,\\ F_{1}\left(\Delta f,C_{j}\right) & \equiv 2\int fG\left(f-C_{1}\right)G\left(f-C_{2}\right)df,\\ L(\Delta f,C_{j}) & \equiv 2\int \lambda \exp\left[i\left(C_{1}-C_{2}\right)\lambda \right]{\left|G\left(\lambda \right)\right|}^{2}. \end{array}$$

For a Gaussian G(f) in Equation (11), as Δ→0 we have

(A6) $$\begin{array}{cc} F_{0}\left(\Delta f,C_{j}\right) & ∼\exp[-{\left(C_{1}-C_{2}\right)}^{2}/2\Delta f^{2}]\to 0,\\ F_{1}\left(\Delta f,C_{j}\right) & ∼|C_{1}-C_{2}|\exp\left[-{\left(C_{1}-C_{2}\right)}^{2}/2\Delta f^{2}\right]\to 0,\\ L(\Delta f,C_{j}) & ∼\frac{|C_{1}-C_{2}|}{\Delta f^{2}}\exp\left[-{\left(C_{1}-C_{2}\right)}^{2}/2\Delta f^{2}\right]\to 0. \end{array}$$
